# The infection of *Harmonia axyridis* by a parasitic nematode is mediated by entomopathogenic bacteria and triggers sex-specific host immune responses

**DOI:** 10.1038/s41598-018-34278-x

**Published:** 2018-10-29

**Authors:** Tobias Gegner, Tessa Carrau, Andreas Vilcinskas, Kwang-Zin Lee

**Affiliations:** 1 Fraunhofer Institute for Molecular Biology and Applied Ecology, Winchester Strasse 2, D-35394 Giessen, Germany; 2 Institute for Insect Biotechnology, Justus-Liebig-University, Heinrich-Buff-Ring 26-32, D-35392 Giessen, Germany

## Abstract

The harlequin ladybird *Harmonia axyridis* is native to Asia but has been introduced into many countries as a biological control agent. It is now considered an invasive pest, threatening the biodiversity of native ladybirds globally, in part because of its superior immune system. *H. axyridis* is infected and killed by the parasitic nematode *Parasitylenchus bifurcatus*, which could therefore be developed as a biological strategy to counter the spread of this insect pest. However, effective control requires an understanding of the tripartite relationship between *H. axyridis*, *P. bifurcatus* and their potential bacterial mutualists. Here we describe the isolation of two species of nematode-associated bacteria (*Serratia marcescens* and *Providencia rettgeri*) which were highly virulent against *H. axyridis* in survival experiments. In addition, contact between the nematodes and beetles led to the sex-specific modulation of multiple host immunity-related genes after 24 and 48 h, with many genes encoding antimicrobial peptides rapidly and stably repressed in females whereas the same genes were initially induced in males before suppression at the later time point. These data provide evidence that the female immune system responds much more strongly to the nematodes and provokes, in turn, a more robust invasion strategy involving the bacterial mutualists.

## Introduction

The harlequin ladybird *Harmonia axyridis* is native to Central and East Asia but has been introduced into many countries to control aphids and other pests^1^. Within a short period, *H. axyridis* has become an invasive species which successfully outcompetes native ladybirds worldwide^1,2^. Therefore, it is now used as a model to determine why some species become successful invaders whereas others, even if closely related, do not^3^. The invasive success of *H. axyridis* can partly be explained by behavioural adaptations such as intraguild predation^4^, as well as the transmission of microsporidia which are tolerated by the host but not by native ladybird species^5,6^. However, other important factors inlcude the superior immune system of *H. axyridis*, which provides stronger resistance against pathogens and parasites compared to native ladybird species such as *Adalia bipunctata* and *Coccinella septempunctata*^7–11^.

*H. axyridis* has a dual defence system comprising the alkaloid harmonine, which is constitutively present in the haemolymph, and inducible antimicrobial peptides (AMPs), which are expressed when pathogens are encountered^12^. Interestingly, harmonine displays broad-spectrum activity against parasites, including those responsible for malaria and leishmaniosis in humans^13,14^. The *H. axyridis* genome contains an expanded repertoire of 49 genes encoding putative AMPs and 10 genes encoding lysozymes, the highest number of defence-related peptides produced by any animal species investigated thus far^15^. Harmonine and AMPs display synergistic antimicrobial activity with c-type lysozymes^16^. The superior immune system of *H. axyridis* may also enable this host to carry the parasitic microsporidia described above, and to use them as biological weapons against competing ladybird species without suffering their effects directly^5,6,17,18^. We recently demonstrated that invasive and non-invasive populations of *H. axyridis* show distinct expression profiles of certain AMPs and confirmed the important protective role of one specific coleoptericin in the invasive populations, which suggests that rapidly-changing gene expression profiles in *H. axyridis* populations can promote pathogen resistance and thereby their invasive performance^19^.

Despite the superior *H. axyridis* immune system, there are several entomopathogenic bacteria^20^, fungi^7–10^ and nematodes^21^ that kill this species. The ability of entomopathogenic nematodes to infect and kill insects^22^ provides an environmentally sustainable alternative to chemical pesticides for biological control^23,24^. A well-studied example is the nematode *Heterorhabditis bacteriophora*, which forms a mutualistic relationship with the highly entomopathogenic Gram-negative bacterium *Photorhabdus luminescens*^25^. The first report of nematodes in ladybirds^26^ revealed a new nematode species later named *Parasitylenchus coccinellinae*^27^. A similar entomopathogenic nematode discovered in *H. axyridis* beetles^28^ was later named *P. bifurcatus*^29^.

The analysis of complex tripartite relationships between insect hosts, parasitic nematodes and their bacterial mutualists has thus far been limited to model species such as *Drosophila melanogaster*^30^ and *Manduca sexta*^31^. Here we investigated the relationship between *H. axyridis*, *P. bifurcatus* and two potential bacterial mutualists of the entomopathogenic nematode, i.e. *Serratia marcescens* and *Providencia rettgeri*. Both bacterial strains can kill *H. axyridis* rapidly in a concentration-dependent manner. Gene expression profiling in ladybirds carrying the entomopathogenic nematode revealed a complex response involving the sex-specific expression of multiple immunity-related genes 24 and 48 h post-infection.

## Results

### Identification of *P. bifurcatus* and infection rates in *H. axyridis*

**Figure 1:**
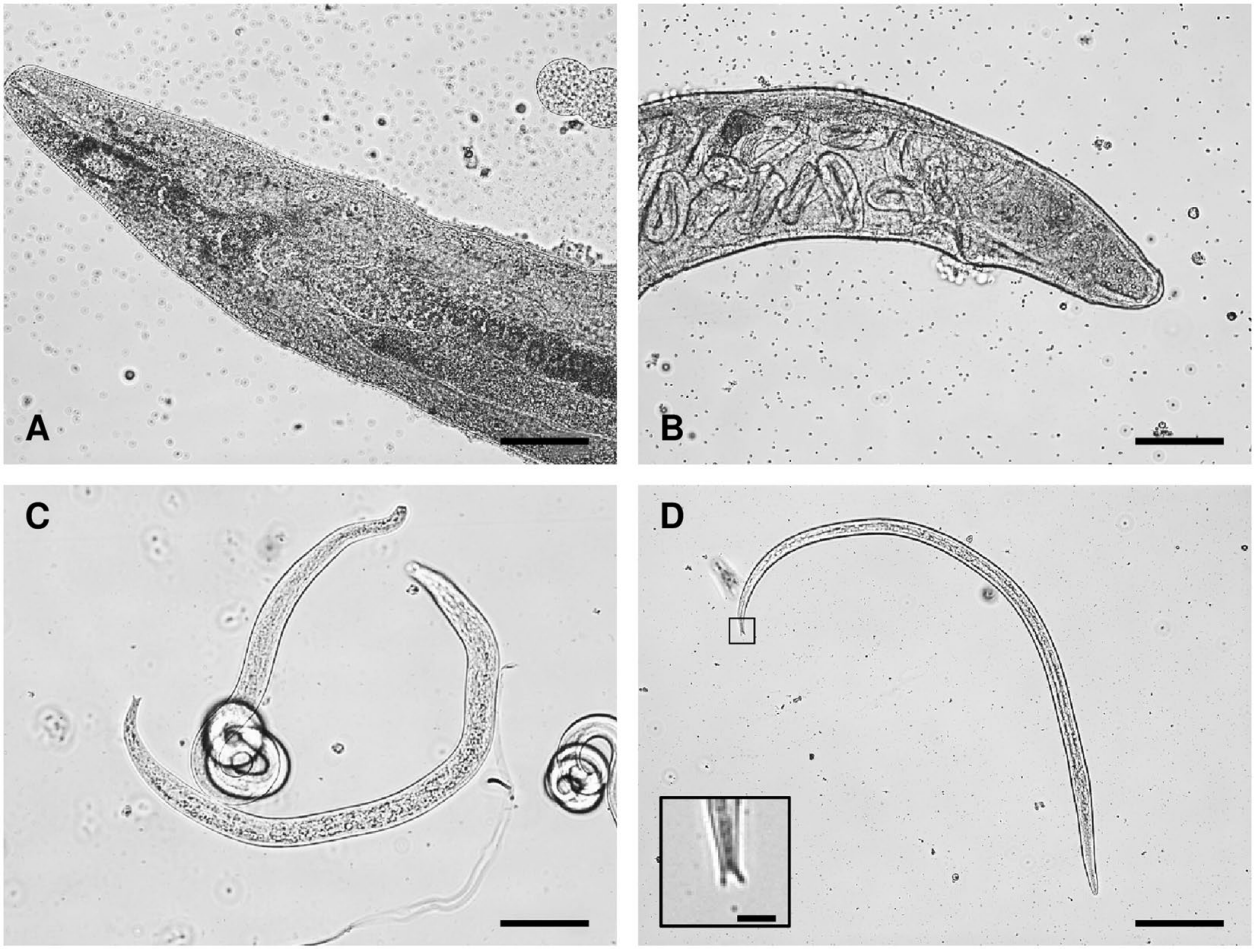
Microscopic images of *P. bifurcatus* isolated from *H. axyridis* beetles. (**A**) Head region. Scale bar = 125 µm. (**B**) Vulva region of mature female. Developing eggs and larvae can be seen in the body cavity. Scale bar = 96 µm. (**C**) Adult male with coiled tail region and juvenile female. Scale bar = 67 µm. (**D**) Infective vermiform female with magnification of the bifurcated tail. Scale bar = 116 µm (main image) and 10 µm (inset image).

Nematodes found in the haemocoel of greenhouse-reared *H. axyridis* beetles were identified morphologically as the previously described species *P. bifurcatus* (Tylenchida: Allantonematidae) (Fig. 1A–D) and were consistent with earlier descriptions^28,29^, including morphological characteristics like a straight stylet lacking basal knobs, a short and narrow bursa of the males and especially the presence of the eponymous bifurcated tail of juvenile male and vermiform female nematodes (Fig. 1D). Phylogenetic identification on the basis of 18*S* SSU rRNA gene sequences confirmed the morphological identification. BLAST search results and the resulting consensus sequence from the assembly are presented in Table S1 and Fig. S1. Field-collected beetles were screened for *P. bifurcatus*, revealing a low prevalence of 1.23% for aggregating individuals in October 2017 (Table 1). In contrast, we found that 75% of the greenhouse-reared beetles were infected with *P. bifurcatus*, but the laboratory-bred specimens as well as L4 larvae from the greenhouse-reared population showed no evidence of nematode infections (Table 1).

**Table 1:** Prevalence of *P. bifurcatus* in field-collected, greenhouse-reared, and laboratory-reared *H. axyridis* beetles and in greenhouse-reared L4 larvae.

	Individuals	Uninfected	%	Infected	%
Beetles, field-collected	324	320	98.77	4	1.23
Beetles, greenhouse-reared	80	20	25.00	60	75.00
Beetles, laboratory-reared	142	142	100.00	0	0.00
L4, greenhouse-reared	80	80	100.00	0	0.00

### Isolation of nematode-associated bacteria

**Figure 2:**
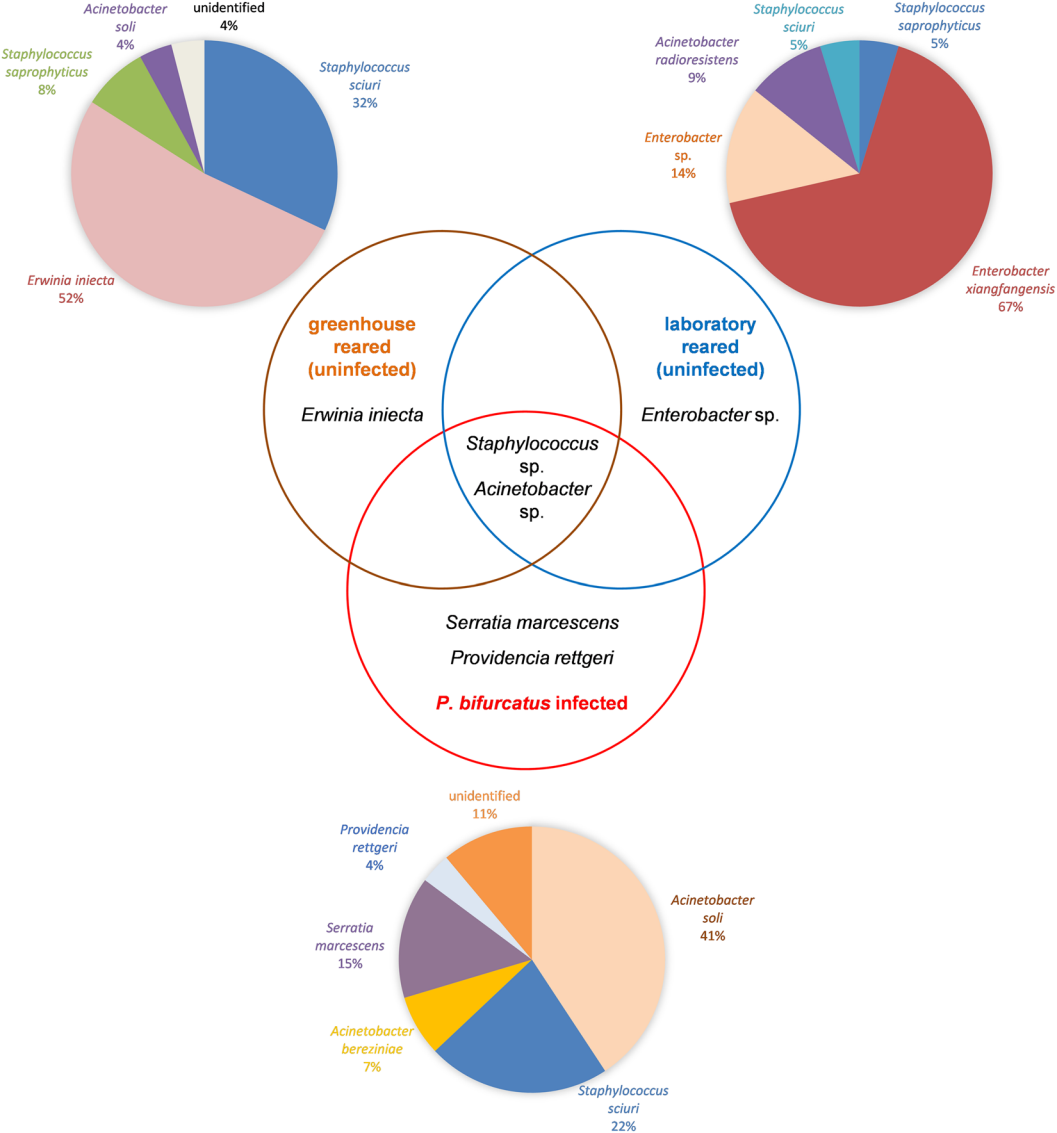
Frequency and distribution of bacterial species found in uninfected *H. axyridis* beetles and those infected with the nematode *P. bifurcatus*. Pie charts indicate species-level composition and frequency in greenhouse-reared (uninfected) beetles based on 21 bacterial sequences, laboratory-reared (uninfected) beetles based on 25 sequences, and nematode-infected beetles based on 27 sequences. Venn diagram shows the shared and exclusive strains (middle).

We established bacterial culture collections from nematode-infected greenhouse-reared beetles as well as control collections from nematode-free greenhouse-reared and laboratory-reared individuals, and identified the bacteria by sequencing the 16*S* SSU rRNA gene (Fig. 2). In the uninfected greenhouse-reared controls, 52% of the 21 sequenced clones were identified as *Erwinia iniecta*, 32% as *Staphylococcus sciuri*, 8% as *Staphylococcus saprophyticus*, 4% as *Acinetobacter soli* and 4% were unidentified. In the uninfected laboratory-reared controls, 67% of the 25 sequenced clones were identified as *Enterobacter xiangfangensis*, 14% as other *Enterobacter* spp., 9% as *Acinetobacter radioresistens*, 5% as *S. sciuri* and 5% as *S. saprophyticus*. In the beetles infected with *P. bifurcatus*, 41% of the 27 sequenced clones were identified as *A. soli*, 22% as *S. sciuri*, 15% as *Serratia marcescens*, 7% as *Acinetobacter bereziniae*, 4% as *Providencia rettgeri* and 11% were unidentified. All three groups carried *S. sciuri* and *Acinetobacter* spp., suggesting these species may be part of the normal *H. axyridis* microbiome. Two species, *S. marcescens* and *P. rettgeri*, were exclusively found in beetles infected with *P. bifurcatus*, indicating a potential mutualistic association with the nematode. BLAST search results and 16*S* SSU rRNA sequences for *S. sciuri*, *S. marcescens* and *P. rettgeri* are presented in Tables S2–S4 and Figs S2–S4.

### Susceptibility of *H. axyridis* to nematode-associated bacteria

**Figure 3:**
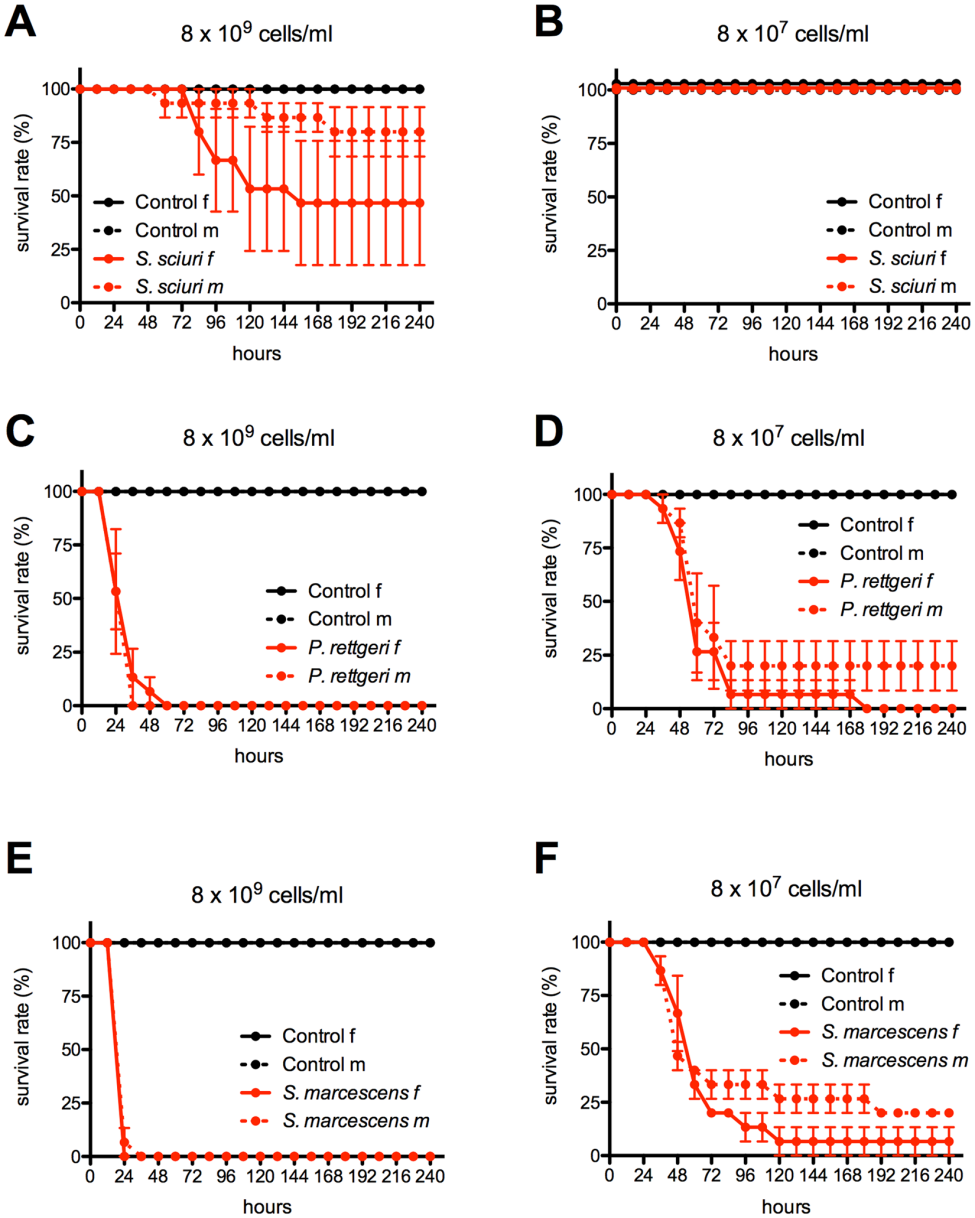
Survival of *H. axyridis* male and female beetles injected with different bacterial isolates compared to control injections with PBS. Left-hand panels (A,C,E) represent bacterial concentrations of 8 × 10^9^ cells/ml, and right-hand panels (B,D,F) represent bacterial concentrations of 8 × 10^7^ cells/ml. (**A,B**) Infection with *S. sciuri* as an internal microbiome control. (**C,D**) Infection with the nematode-associated *P. rettgeri* strain. (**E,F**) Infection with the nematode-associated *S. marcescens* strain. Error bars indicate standard deviation of the mean of three independent experiments (3 × 5 beetles per sex and treatment).

The virulence of the nematode-specific bacterial strains *S. marcescens* and *P. rettgeri*, and *S. sciuri* as an internal microbiome control, was determined by infecting beetles via intra-thoracic injection at the third coxal base with either a bacterial suspension or phosphate-buffered saline (PBS) as mock control. Two different concentrations of bacteria, adjusted with a Neubauer improved counting chamber, were tested: 8 × 10^7^ and 8 × 10^9^ cells/ml (Fig. 3A–F). Survival was recorded every 12 h up to 240 h. All PBS-injected controls showed 100% survival at 240 h, i.e. 10 days after infection (Fig. 3A–F). The beetles injected with *S. sciuri* at the higher concentration of 8 × 10^9^ cells/ml showed a low male mortality and intermediate female mortality with a median survival time (ST_50_) of 156 h (95% CI, 96–NA) (Fig. 3A), which was significantly different from PBS controls (log-rank test: p < 0.001) but not from infected males (log-rank test: p = 0.0539). Mortality after *S. sciuri* infection was not evident at the lower concentration of 8 × 10^7^ cells/ml (Fig. 3B). In contrast, survival of beetles injected with either concentration of *P. rettgeri* or *S. marcescens* always was lower when compared to controls and *S. sciuri* (log-rank test: p < 0.001 each). *P. rettgeri* rapidly killed all the beetles at the higher concentration of 8 × 10^9^ cells/ml, with a ST_50_ of 36 h (95% CI, 24–36) and 100% mortality after 60 h (Fig. 3C). At the lower concentration of 8 × 10^7^ cells/ml there was a concentration dependent shift in survival, with a ST_50_ of 60 h (95% CI, 60–84) and ~20% of the males remained alive 240 h post infection (Fig. 3D). Though, all injected females died within 180 h, the difference between sexes was not significant (log-rank test: p = 0.1713). Similar results were observed when the beetles were injected with *S. marcescens*. At the higher concentration, all beetles succumbed within 24 h (females) and 36 h (males), respectively (Fig. 3E), showing that *S. marcescens* is even more virulent than *P. rettgeri* (log-rank test: p = 0.0021 and p = 0.0120). At the lower concentration, no difference in virulence was observed between those two pathogens (log-rank test: p = 1.000 for both sexes) and the ST_50_ for *S. marcescens* was similar to *P. rettgeri* with approximately 60 h (95% CI, 60–72) (Fig. 3F). Statistical summaries including values for ST_50_, log-rank tests for both bacterial concentrations and pairwise comparisons between sexes with corresponding p-values can be found in Tables S5–S7.

### AMP gene expression in nematode-infected beetles

**Figure 4:**
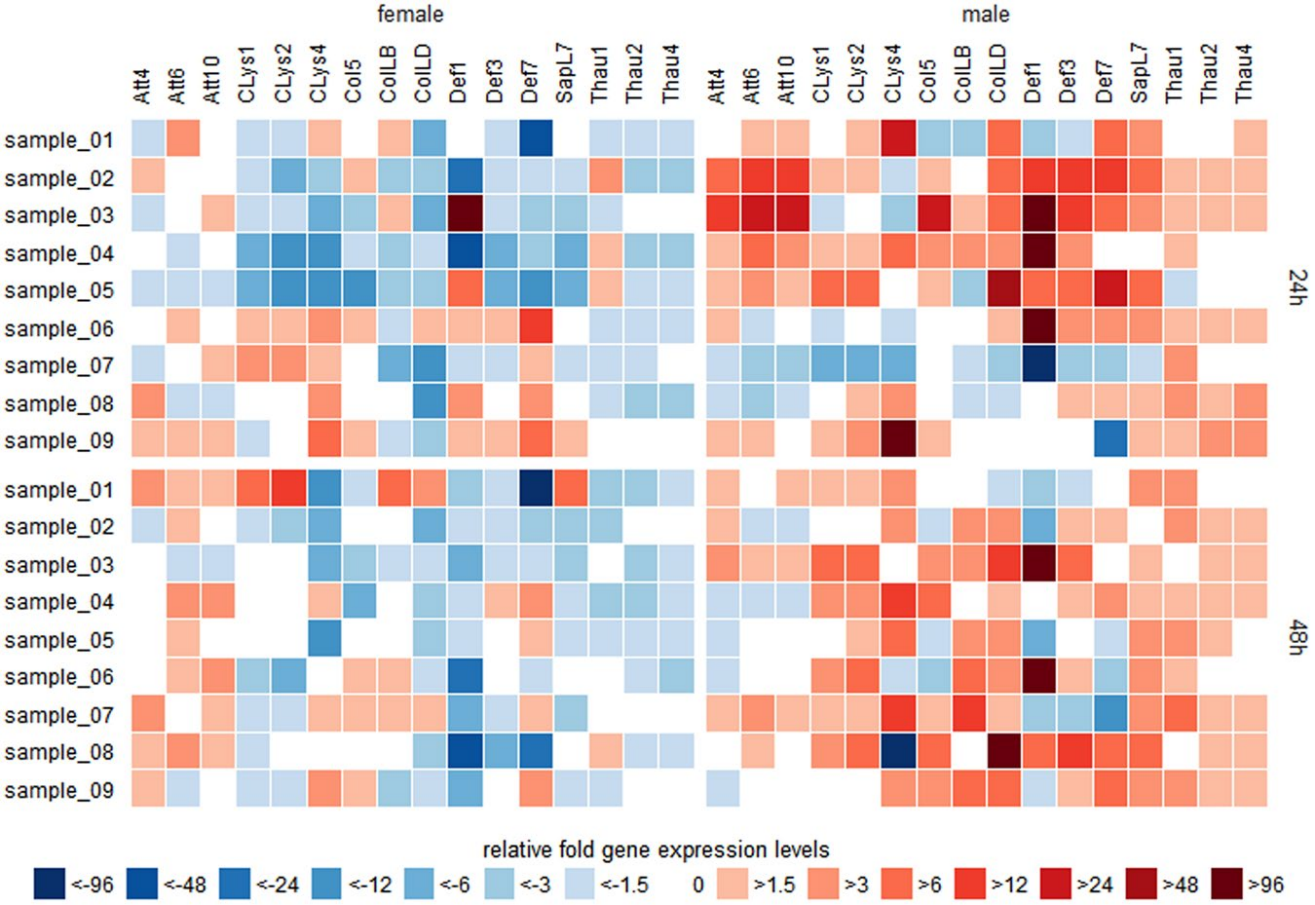
Analysis of the *P. bifurcatus* induced sex-specific AMP gene expression in *H. axyridis*. The heat map shows the comparison of immunity-related genes (listed along the top) regulated in the presence of *P. bifurcatus* between females (left panel column) and males (right panel column) after 24 h (upper panel row) and 48 h (lower panel row). Each sample represents the relative fold-change in gene expression for each infected beetle (n = 9 per sex and time point) against its corresponding mean control value (n = 3 per experiment and time point). The colour code reflects the magnitude of fold-induction or -repression compared to uninfected controls. The 24-h samples were sorted separately for each sex by hierarchical agglomerative cluster analysis using Euclidean distance matrix computation and Ward’s minimum variance algorithm and the resulting order was also used for the 48-h samples.

Next we analysed the transcriptional modulation of 22 genes encoding AMPs in beetles exposed to nematodes carrying the two bacterial strains described above (Fig. 4). Each sample used for quantitative real-time PCR analysis represents an individual beetle dipped in PBS containing nematodes to initiate infection via the natural exposure route (infected beetle expression value) paired with the mean value of six naïve controls dipped in PBS (mean control expression value). The induction ratio was categorized at 15 different levels and the results are presented as a heat map, which compares the relative fold-changes in gene expression between nine infected females and nine infected males at 24 and 48 hours after infection.

Overall, we observed substantial differences in relative immunity-related gene expression between males and females at both time points, whereas the changes from 24 to 48 h post-infection were less pronounced. After 24 h (Fig. 4, upper panel row), many genes in the females (Fig. 4, left column) were downregulated compared to controls and only a few genes were induced. Females 2 and 4 shared the overall repression of almost all AMPs except Thau1, which was consistently induced from 1.5-fold to 6-fold. Females 3 and 5 showed an overall expression profile similar to females 2 and 4, but differed in their strong induction of Def1, which they shared with males 2–6. Females 6–9 shared a common profile, featuring the consistent downregulation of thaumatins and the low to moderate induction of attacins, c-type lysozymes and defensins. In particular these samples revealed the consistent upregulation of CLys4 and Def4 from 1.5-fold to 12-fold and 24-fold, respectively. In contrast to the female samples, males (Fig. 4, right column) showed the moderate to strong induction of many AMP genes, especially those encoding attacins, defensins and some coleopterins and c-type lysozymes. Thaumatins were also moderately upregulated (up to 6-fold) in all males, except male 5. Only male 7 showed an expression profile differing from the other males, and like females 2, 4 and 5 this profile featured the general downregulation of AMPs and moderate upregulation of Thau1. Males 1, 8 and 9 showed a similar induction of c-type lysozymes, especially CLys4, with relative expression changes from 3-fold to 102-fold. Males 2–6 showed a very strong induction of Def1 (8240-fold in male 3) as well as the moderate induction of other defensins (including SapL7) and attacins.

The expression profiles after 48 h (Fig. 4, lower panel row) were similar to those observed at 24 h, so the major differences we observed were sex-dependent rather than time-dependent. The expression profiles were particularly stable in the female samples (Fig. 4, left column). The most remarkable differences were the declining induction ratio of Thau1 in females 2, 4 and 5, the moderate upregulation of Att6 and the switch from strong upregulation to repression of Def1 in females 3 and 5, and the declining induction ratio of c-type lysozymes and defensins in females 6–9. There were more substantial changes in gene expression between 24 and 48 h in the males, but the expression of thaumatins remained stable (Fig. 4, right column). We observed the moderate induction of attacins, c-type lysozymes, coleoptericins and thaumatins in males 7 and 8, leading to similar expression profiles as observed in most males at 24 h. In males 2–6, the induction ratio of the attacins and defensins declines, with the exception of SapL7. Furthermore, with the exception of males 3 and 6, we found that the formerly strongly induced Def 1 was downregulated at 48 h compared to controls. Overall, our analysis showed that after *P. bifurcatus* challenge in *H. axyridis* the host’s immune related genes are differentially expressed between females and males.

## Discussion

The invasive ladybird *H. axyridis* has a robust and highly adjustable immune system which may contribute to its invasive success by conferring resistance against pathogens and parasites encountered in newly colonized habitats^11,15,19,32^. We found that our greenhouse-reared *H. axyridis* population was infected with the entomopathogenic nematode *P. bifurcatus*, which was previously shown to be carried by its host with a prevalence of 2–33% in early autumn^28,29^, consistent with the prevalence of 1.23% we observed among 324 field-collected beetles. We also observed a prevalence of 75% for greenhouse-reared beetles but no infections in laboratory-reared beetles, indicating an absence of vertical transmission from greenhouse-reared beetles to F1 eggs and larvae. This also explains why individual laboratory rearing provided us with 100% nematode free beetles for our experiments. Furthermore, our data for greenhouse reared L4 larvae (Table 1), which were found to be void of nematodes, support the hypothesis that the complete life cycle of *P. bifurcatus* is restricted to adult *H. axyridis*^29^. The route of transmission is currently unknown, but is likely to involve orifices such as the mouth, anus, spiracles and tracheae, or through soft parts of the cuticle^33^.

Entomopathogenic nematodes are parasites that kill insects^22^. However, the high prevalence of *P. bifurcatus* in greenhouse-reared *H. axyridis* did not lead to a decline in the population. Although the nematodes are not immediately lethal to their host, they are likely to reduce host fitness by depleting nutritional reserves^33^. Entomopathogenic nematodes are associated with bacteria that are virulent in the insect host, such as nematodes of the genera *Steinernema* and *Heterorhabditis* which carry mutualistic bacteria of the genera *Xenorhabdus* and *Photorhabdus*, respectively^34^. We therefore screened our nematode-infected beetles for bacteria that are not found in nematode-free specimens, and identified the Gram-negative species *S. marcescens* and *P. rettgeri*.

*Serratia marcescens* has a wide host range including model nematodes such as *Caenorhabditis elegans*, and it is considered an efficient killer^35,36^. However, the newly recognized *Serratia* isolate SCBI (South African *Caenorhabditis briggsae* isolate) is non-pathogenic in the *Caenorhabditis briggsae* strain KT0001^37,38^. In this context, the nematode and bacteria may enjoy a mutualistic relationship in which the bacteria confer new virulence traits upon their nematode host in exchange for the ability to establish an environmental niche, as shown by the inability of nematodes to kill the greater wax moth *Galleria mellonella* when *S. marcescens* is replaced by the non-pathogenic *Escherichia coli* strain OP50^37^. We tested the virulence of our newly isolated strain of *S. marcescens* and found that *H. axyridis* was extremely susceptible, suggesting that a similar mutualistic relationship may have been established between *S. marcescens* and *P. bifurcatus*. Future work with infection experiments using axenic nematodes is needed to delineate the role of the mutualistic bacteria, or the role of nematode alone in the infection model, respectively.

*Providencia rettgeri* is a Gram-negative bacterium commonly found in water and land environments, although strains of this species have also been isolated from nematodes of the genus *Heterorhabditis*^39^. Again, these bacteria are highly virulent in *G. mellonella* larvae. Another *Providencia* species isolated from the entomopathogenic nematode *Steinernema thermophilum* is similar to *P. rettgeri* but was identified as a new strain of *Providencia vermicola*^40^. Our phylogenetic analysis based on 16*S* SSU rRNA sequences identified our *Providencia* strain as *P. rettgeri* (Fig. S5). Like the *S. marcescens* strain described above, the new *P. rettgeri* strain was found to be highly virulent towards *H. axyridis*.

It is surprising that the direct infection of *H. axyridis* with *S. marcescens* or *P. rettgeri* resulted in rapid death, whereas the infection of *H. axyridis* with *P. bifurcatus* carrying both bacteria is consistent with the maintenance of a stable ladybird population. The great potential of these two bacteria to kill *H. axyridis* becomes even more visible when compared to the virulence of the well-known entomopathogenic bacterium *Pseudomonas entomophila*. In our previous study^19^, *P. entomophila* reduced host survival to ~50% after 10 days, whereas similar concentrations (8 × 10^7^ cells/ml) of *S. marcescens* and *P. rettgeri* in the current study reduced host survival to 13% and 10%, respectively, with a ST_50_ of 60 h (95% CI, 60–72 and 60–84) and a trend for males possibly being less susceptible. This may indicate that the bacteria are not efficiently released from the nematode, or that bacterial growth and virulence are suppressed by the presence of the nematode until a condition is satisfied or signal received that allows their release and/or activation^39^. Symbiosis between entomopathogenic nematodes and more than one bacterial species is rare, increasing the complexity of the interaction network between the host, nematode and bacteria^39,41,42^.

We analysed the immune response of *H. axyridis* when exposed to *P. bifurcatus* by comparing the induction of 22 genes encoding selected AMPs and lysozymes 24 and 48 h after contact with the nematode. This is an important consideration in the context of the more general interrelationship between the host, parasites and mutualistic bacteria because parasitic microsporidia that are naturally carried by *H. axyridis*^5,6,18^ are thought to be suppressed by the presence of harmonine, whose synthesis is downregulated when AMP genes are induced^12^. Our data suggest that infection with *P. bifurcatus* is detected by the *H. axyridis* immune system, resulting in the induction of many AMP genes after 24 h, particularly in males. In *D. melanogaster*, AMPs are induced by axenic nematodes but suppressed in the presence of mutualistic bacteria^43^. These findings suggest that nematodes activate the humoral immune response in *D. melanogaster*, but the bacteria they carry are capable of reversing this change. A similar result was observed with the nematode *Heterorhabditis bacteriophora* and its mutualistic bacterium *Photorhabdus temperata* in the tobacco hornworm *Heliothis virescens*^44^. Initial penetration by the nematode caused the strong induction of host immunity-related genes, but the same genes were suppressed following the release of the bacteria into the haemolymph.

In light of those results we propose an analogous mode of action in our model, where AMP expression is induced by *P. bifurcatus* infection, but supressed earlier in females than males by entomopathogenic mutualists carried by the nematodes. Given that all the samples were treated in the same manner, our results suggest there may be a variable timescale between the induction of gene expression by the nematodes and the subsequent suppression caused by the bacteria, probably reflecting a long ‘infection window’ with infection becoming established in different beetles at different times. Females, for example, show the repression of almost all of the AMP genes we tested at 24 h, indicating that the infection was established quickly and that the nematode-specific induction may have been overcome by the bacterial suppression before we took the first measurements. In contrast, male samples 1 and 2 show low AMP induction at 24 h, but moderate to high induction in many genes after 48 h, suggesting the nematode induction phase may have lasted longer in these individuals. One explanation is that, according to Bateman’s principle and its implications for the insect immune system^45^, females often show stronger immune responses than males. Therefore nematodes might be tackled earlier in females and find it more difficult to establish an infection, which causes them to release their mutualists earlier to supress the strong innate immune defences. Because both *Providencia rettgeri* and *Serratia marcescens* rapidly and efficiently killed the beetles in our survival experiments, this might also explain the rapid suppression of AMPs.

To summarize, our AMP gene expression analysis revealed a complex set of gene expression profiles that are likely to depend on the specific characteristics of each infection and the infection time. Nevertheless, we have analyzed the expression profiles of AMP genes that seem to be affected by nematode infection and potentially by subsequent mutualist suppression, resulting in a much stronger immune response in male than female *H. axyridis* beetles. A direct bacterial inhibition of the host AMP transcription, as described for *Shigella spp*. evasion in mammalian host^46,47^ has so far not been shown for *P. rettgeri*. In contrast, the *S. marcescens* cyclic depsipeptide AT514 has been shown to decrease NF-κB activity, responsible transcription factor for AMP expression, in a B-cell chronic lymphocytic leukemia model^48^. Other bacterial mechanisms to control the host include the inhibition of cellular defences, for example the *S. marcescens* metalloprotease serralysin that displayed inhibition of immune cell adhesion and bacterial clearance in the silkworm *Bombyx mori*^49^. Our results further support the hypothesis advanced in our previous study that rapid changes in gene expression and a highly adjustable immune system, consisting of the constitutively produced defense compound harmonine and a broad repertoire of inducible AMPs, could promote pathogen resistance and consequently the invasiveness of *H. axyridis* populations^19^. Here we extend this hypothesis by proposing the underlying principles are especially relevant for *H. axyridis* females: the sex-dependent AMP gene expression profiles observed after infection with *P. bifurcatus* carrying entomopathogenic mutualists reveal that the nematodes need to suppress the female immune system much more rapidly and vigorously than in males. In perspective, further studies using a defined *in vitro* infection model, *i.e*. the possibility of raising nematodes on a defined media, controlled infection with a defined number of nematodes and further characterization of the identified bacterial strains, including the assessment of bacterial load after infection, are required to elucidate the interaction between host, nematode and bacteria. The ability to genetically modify the identified *S. marcescens* and *P. rettgeri* strains would facilitate the creation of axenic nematodes as described for the generation of axenic *Heterorhabditis* nematodes using a genetic modified *Photorhabdus* strain RET16 with a traceable antibiotic resistance and the inability to colonize the nematode^30^. Future work using axenic nematodes will help to delineate the role of the mutualistic bacteria, or the role of nematode alone in the infection model, respectively.

Thus far, no mutualistic bacteria has been described for the entomopathogenic nematode *P. bifurcatus*, and the identification of the novel nematode associated *Serratia* and *Providencia* strains with their high virulence against the invasive ladybird *H. axyridis* opens the possibilities to develop strategies for biological safe and effective control of this insect, which is a serious invasive pest.

## Materials and Methods

### Collection and rearing of *H. axyridis*

*H. axyridis* individuals were collected in and around Giessen/Ober-Mörlen, Germany, in 2013 and maintained at 21 °C and 60% humidity with a 16-h photoperiod in a greenhouse. Bean plants (*Phaseolus vulgaris*) infested with pea aphids (*Acyrthosiphon pisum*) were provided *ad libitum* as a food source. New specimens were integrated regularly into this greenhouse-reared population in autumn and spring. Experiments were carried out using laboratory-reared beetles that were 2–3 weeks old. Larvae were separated after hatching and reared individually in 35 × 10 mm Petri dishes with the *ad libitum* provision of water and sterile freeze-killed eggs of the grain moth *Sitotroga cerealella* (Katz Biotech AG).

### Nematode screening and species identification

Adult beetles and L4 larvae from the greenhouse-reared population (n = 80 each), as well as laboratory-reared beetles (n = 142) and individuals collected around Ober-Mörlen in late October 2017 (n = 324) were dissected immediately after sampling. Nematodes from infected ladybirds were collected in PBS and the species identified using a FLoid® Cell Imaging Station (Thermo Fisher Scientific) to capture images at different life stages. The nematodes were centrifuged at 16,000 × *g* for 15 min at 4 °C and the pellet was resuspended in nuclease-free water before lysis and amplification of the 18*S* SSU rRNA gene as previously described^50^ using GoTaq® G2 DNA Polymerase (Promega). The PCR products were purified and transferred to the vector pGEM®-T Easy (Promega) for sequencing, followed by sequence analysis using Geneious v10.2.2^51^. A consensus sequence was generated by the *de novo* assembly of the three overlapping sequence fragments and a BLAST search against the NCBI nr/nt database was carried out using Megablast with standard parameters.

### Bacterial isolates and infection experiments

Nematode-associated bacteria were identified by comparing the bacterial communities of *H. axyridis* beetles from three different rearing groups: nematode-free greenhouse-reared, laboratory-reared, and nematode-infected greenhouse-reared beetles. For each rearing group, three beetles were surface sterilised with 80% ethanol and 0.3% bleach, and dissected individually in sterile PBS. Body fluids and/or nematodes in PBS were transferred to individual reaction tubes and centrifuged briefly before homogenization with three 2.3-mm zirconium/glass beads (BioSpec Products) in a FastPrep-24™ 5G instrument (MP Biomedical) at 10 m/s for two periods of 45 s. We then transferred 100-µl extracts onto lysogeny broth agar plates and incubated them at 37 °C for 2 days. We amplified the 16*S* SSU rRNA gene from a total of 90 colonies (ten colonies per beetle sample) using the universal 5′ primer 27F (5′-AGA GTT TGA TCM TGG CTC AG-3′) and universal 3′ primer 1492R (5′-CGG TTA CCT TGT TAC GAC TT-3′). The positive PCR products (from 73 colonies) were treated with Exonuclease I (New England Biolabs) and Shrimp Alkaline Phosphatase (Sigma-Aldrich) for enzymatic removal of excess nucleotides and primers prior to direct sequencing and the obtained sequences were analysed as described above.

### Survival analysis

Overnight cultures of *S. sciuri*, *P. rettgeri* and *S. marcescens* were diluted to 8 × 10^9^ or 8 × 10^7^ cells/ml in PBS using a Neubauer improved haemocytometer (Paul Marienfeld) and 4 µl of bacterial suspension was injected into each beetle as previously described^19^. Control beetles were injected with an equal volume of PBS. After injection, the specimens were kept individually in 35 × 10 mm Petri dishes and provided *ad libitum* with water and *S. cerealella* eggs. Five male and five female individuals were used for each treatment and the experiment was carried out three times (n = 15 for each sex) per concentration. Survival was recorded every 12 h until 240 h (10 days) post-injection.

### AMP gene expression analysis

Immune responses in *H. axyridis* were triggered by dipping beetles in suspensions of *P. bifurcatus* in PBS or PBS only as a control. The presence of the *S. marcescens* and *P. rettgeri* strains in the *P. bifurcatus* suspension was tested as described previously in “bacterial isolates and infection experiments” section. Each nematode suspension (approximately 6000 nematodes/ml, see Fig. S6) was obtained from six infected beetles by dissecting the surface sterilised beetles in sterile PBS and collecting the nematode solutions in reaction tubes. For each time point (24 and 48 h) we dipped three beetles per sex in nematode suspension and three beetles per sex in PBS solution. The experiment was carried out three times (n = 9 per sex, time point and treatment) using fresh nematode and PBS solutions per repeat. The beetles were kept as described above until they were frozen individually in liquid nitrogen after 24 and 48 h and stored at −80 °C. These time points were selected according to knowledge from literature showing that bacteria from entomopathogenic nematodes, like *H. bacteriophora*, rapidly kill hosts within 48 h after nematode penetration^25^ and that antimicrobial activity in insect haemolymph rapidly increases within the first 48 h after infection^30,52^. Total RNA was isolated from individual beetles with TRI-Reagent® (Zymo Research) according to the manufacturer’s instructions. The quantity of RNA was determined using a Nanodrop ND-1000 spectrophotometer (Thermo Fisher Scientific) and cDNA was generated using the iScript cDNA Synthesis Kit (Bio-Rad). The cDNA was amplified by real-time quantitative PCR on a StepOnePlus™ Real-Time PCR System (Applied Biosystems) with a total reaction volume of 10 μl, using 5 μl 2x SensiMix™ SYBR® No-Rox (Bioline), 1 μl cDNA (corresponding to 50 ng RNA) and 250 nM of each AMP-specific primer^19^ (see also Table S8). Non-template reaction mixtures for each gene were run as negative controls on each 96-well plate. The temperature profile consisted of the following steps: initial activation at 95 °C for 15 min, 40 cycles of denaturation at 95 °C for 15 s, annealing at 55 °C for 15 s, and extension at 72 °C for 15 s. A subsequent dissociation curve analysis with 0.5 °C increments from 65 °C to 95 °C was used to verify specific amplification. We averaged the results of two technical replicates per sample and data were normalized using the ribosomal protein gene *RPS3*. Primer efficiency for all genes was estimated to be close to one and similar to the internal control gene RPS3, as they show similar shaped curves in the logarithmic PCR amplification plots recorded by the StepOnePlus Software. This is a valid method for efficiency determination described in literature when analysing many genes simultaneously^53^. In accordance with these assumptions the relative gene expression levels were calculated using the comparative ΔΔCT method^53,54^.

### Statistical analysis

Statistical analysis was carried out using *R* v3.3.3^55^. Control data for the AMP gene expression analysis experiments were compared between experimental repeats for each gene and time point^19^. Briefly, a parametric multiple comparison analysis^56^ was used on a one-way ANOVA model with ΔCT as the response variable and replicate as the factor to test for pairwise differences (Table S9). The p-values were adjusted using the heteroscedasticity robust sandwich estimator for the covariance matrix^57,58^. AMP genes with controls showing significant differences in expression (Att18, CLys3, Col1, Col8, ColLA, ColLC) were excluded from the analysis. To further minimize differences, PBS controls were averaged per experiment and the relative gene expression levels (2^ΔΔCT^) of each infected individual were calculated with the corresponding mean control value. The quantitative PCR results were then assigned to 15 categories according to the magnitude of the fold-changes for each gene: category 1: <−96, category 2: <−48, category 3: <−24, category 4: <−12, category 5: <−6, category 6: <−3, category 7: <−1.5, category 8: 0, category 9: >1.5, category 10: >3, category 11: >6, category 12: >12, category 13: >24, category 14: >48, and category 15: >96. For 2^ΔΔCT^ values between zero and one, we calculated the negative inverse by dividing −1 with 2^ΔΔCT^, which expresses downregulations as fold change reductions in gene expression^53^. A heat map was plotted to compare the fold-change in gene expression between females and males at 24 and 48 h. Samples at 24 hpi were sorted for each sex by hierarchical agglomerative cluster analysis using Euclidean distance matrix computation and Ward’s minimum variance algorithm, as implemented in *R* using the functions *dist* and *hclust*^59^. The resulting order was also used for the samples at 48 h to facilitate the comparison of relative gene expression between females and males at both time points. Differences in survival rates were analysed using the *R* package survival v2.40-1^60^ with the log-rank test (Kaplan-Meier) and Holm-corrected p-values.

## Electronic supplementary material

Supplementary Information

Dataset S1

Dataset S2

## Data Availability

All data are accessible in the Supplementary Information.
