# Supplementary material for: The infection of Harmonia axyridis by a parasitic nematode is mediated by entomopathogenic bacteria and triggers sex-specific host immune responses
Source: Sci Rep. 2018 Oct 29;8:15938. doi: 10.1038/s41598-018-34278-x (PMC6206131; doi:10.1038/s41598-018-34278-x)

Supplementary information to the manuscript

**The infection of *Harmonia axyridis* by a parasitic nematode is mediated by entomopathogenic bacteria and triggers sex-specific host immune responses**

Tobias Gegner^a^, Tessa Carrau^a^, Andreas Vilcinskas^a,b^ and Kwang-Zin Lee^a,b*^

^a^ Fraunhofer Institute for Molecular Biology and Applied Ecology, Winchester Strasse 2, D-35394 Giessen, Germany

^b^ Institute for Insect Biotechnology, Justus-Liebig-University, Heinrich-Buff-Ring 26-32, D-35392 Giessen, Germany

* Corresponding author, email: kwang-zin.lee@agrar.uni-giessen.de

**Supplementary Files**

File S1 qPCR data for AMP gene expression analysis

File S2 Survival data

**Supplementary Tables and Figures**

**Table S1.** The 20 best hits from a BLAST search using the *Parasitylenchus bifurcatus* consensus partial 18*S* SSU rRNA gene sequence based on the *de novo* assembly of the overlapping PCR products.

| **Accession** | **Bit-Score** | **E Value** | **Organism** | **Sequence Length** | **% Pairwise Identity** | **% Identical Sites** | **% Query coverage** |
| --- | --- | --- | --- | --- | --- | --- | --- |
| LT547722 | 3206.9 | 0 | *Parasitylenchus bifurcatus* | 1741 | 99.90 | 99.90 | 100.00 |
| LT547721 | 3206.9 | 0 | *Parasitylenchus bifurcatus* | 1741 | 99.90 | 99.90 | 100.00 |
| LT547723 | 3190.28 | 0 | *Parasitylenchus bifurcatus* | 1741 | 99.70 | 99.70 | 100.00 |
| LT547719 | 3190.28 | 0 | *Parasitylenchus bifurcatus* | 1741 | 99.70 | 99.70 | 100.00 |
| LT547724 | 3184.74 | 0 | *Parasitylenchus bifurcatus* | 1741 | 99.70 | 99.70 | 100.00 |
| LT547720 | 3184.74 | 0 | *Parasitylenchus bifurcatus* | 1741 | 99.70 | 99.70 | 100.00 |
| KC875397 | 3140.42 | 0 | *Parasitylenchus bifurcatus* | 1702 | 100.00 | 99.90 | 97.76 |
| KC875401 | 3134.88 | 0 | *Parasitylenchus bifurcatus* | 1702 | 99.90 | 99.90 | 97.76 |
| KC875400 | 3129.34 | 0 | *Parasitylenchus bifurcatus* | 1702 | 99.90 | 99.80 | 97.76 |
| KC875399 | 3123.8 | 0 | *Parasitylenchus bifurcatus* | 1702 | 99.80 | 99.80 | 97.76 |
| KC875398 | 3118.26 | 0 | *Parasitylenchus bifurcatus* | 1702 | 99.70 | 99.70 | 97.76 |
| LT629306 | 2994.54 | 0 | *Parasitylenchus bifurcatus* | 1623 | 100.00 | 99.90 | 93.22 |
| LT629307 | 2989 | 0 | *Parasitylenchus bifurcatus* | 1623 | 99.90 | 99.90 | 93.22 |
| LT547725 | 2989 | 0 | *Parasitylenchus bifurcatus* | 1623 | 99.90 | 99.90 | 93.22 |
| LT547726 | 2983.46 | 0 | *Parasitylenchus bifurcatus* | 1623 | 99.80 | 99.80 | 93.22 |
| FJ004889 | 2636.29 | 0 | *Deladenus siricidicola* | 1734 | 94.20 | 94.20 | 99.48 |
| LC147027 | 2588.28 | 0 | *Tylenchomorpha sp.* | 1718 | 94.00 | 93.90 | 98.56 |
| EU545475 | 2579.04 | 0 | *Deladenus siricidicola* | 1709 | 94.00 | 94.00 | 97.99 |
| AF519233 | 2551.34 | 0 | *Howardula sp.* | 1694 | 93.90 | 93.90 | 97.19 |
| FJ004890 | 2542.11 | 0 | *Deladenus siricidicola* | 1674 | 94.20 | 94.10 | 96.04 |

**Table S2.** The 20 best hits from a BLAST search using the *Staphylococcus sciuri* consensus sequence of clones 60ED58, 60ED89 and 60EI10 (partial 16*S* SSU rRNA gene sequence).

| **Accession** | **Bit-Score** | **E Value** | **Organism** | **Sequence Length** | **% Pairwise Identity** | **% Identical Sites** | **% Query coverage** |
| --- | --- | --- | --- | --- | --- | --- | --- |
| MF801325 | 1735.12 | 0 | *Staphylococcus sciuri* | 939 | 100.00 | 100.00 | 100.00 |
| KY653115 | 1735.12 | 0 | *Staphylococcus sciuri* | 939 | 100.00 | 100.00 | 100.00 |
| KY608160 | 1735.12 | 0 | *Staphylococcus lentus* | 939 | 100.00 | 100.00 | 100.00 |
| KX344009 | 1735.12 | 0 | *Staphylococcus sciuri* | 939 | 100.00 | 100.00 | 100.00 |
| KX280779 | 1735.12 | 0 | *Staphylococcus sciuri* | 939 | 100.00 | 100.00 | 100.00 |
| KX023250 | 1735.12 | 0 | *Staphylococcus sciuri* | 939 | 100.00 | 100.00 | 100.00 |
| KU867651 | 1735.12 | 0 | Bacterium strain | 939 | 100.00 | 100.00 | 100.00 |
| KU863621 | 1735.12 | 0 | Bacterium strain | 939 | 100.00 | 100.00 | 100.00 |
| KU377302 | 1735.12 | 0 | *Staphylococcus sciuri* | 939 | 100.00 | 100.00 | 100.00 |
| KU245713 | 1735.12 | 0 | *Staphylococcus sp.* | 939 | 100.00 | 100.00 | 100.00 |
| KT260686 | 1735.12 | 0 | *Staphylococcus sciuri* | 939 | 100.00 | 100.00 | 100.00 |
| KR186179 | 1735.12 | 0 | *Staphylococcus sciuri* | 939 | 100.00 | 100.00 | 100.00 |
| KM370128 | 1735.12 | 0 | *Staphylococcus sciuri* | 939 | 100.00 | 100.00 | 100.00 |
| KJ000305 | 1735.12 | 0 | *Staphylococcus sciuri* | 939 | 100.00 | 100.00 | 100.00 |
| KJ000304 | 1735.12 | 0 | *Staphylococcus sciuri* | 939 | 100.00 | 100.00 | 100.00 |
| KJ000302 | 1735.12 | 0 | *Staphylococcus sciuri* | 939 | 100.00 | 100.00 | 100.00 |
| JX871318 | 1735.12 | 0 | *Staphylococcus sciuri* | 939 | 100.00 | 100.00 | 100.00 |
| JX134627 | 1735.12 | 0 | *Staphylococcus sciuri* | 939 | 100.00 | 100.00 | 100.00 |
| JX077096 | 1735.12 | 0 | *Staphylococcus sciuri* | 939 | 100.00 | 100.00 | 100.00 |
| JQ975877 | 1735.12 | 0 | Endosymbiont of *Nilaparvata lugens* | 939 | 100.00 | 100.00 | 100.00 |

**Table S3.** The 20 best hits from a BLAST using the *Serratia marcescens* consensus sequence of clones 60ED57, 60EI15 and 61CD07 (partial 16*S* SSU rRNA gene sequence).

| **Accession** | **Bit-Score** | **E Value** | **Organism** | **Sequence Length** | **% Pairwise Identity** | **% Identical Sites** | **% Query coverage** |
| --- | --- | --- | --- | --- | --- | --- | --- |
| MG571677 | 1679.72 | 0 | *Serratia marcescens ssp. sakuensis* | 909 | 100.00 | 100.00 | 100.00 |
| MG491661 | 1679.72 | 0 | *Serratia sp.* | 909 | 100.00 | 100.00 | 100.00 |
| MG491656 | 1679.72 | 0 | *Serratia sp.* | 909 | 100.00 | 100.00 | 100.00 |
| MG491655 | 1679.72 | 0 | *Serratia sp.* | 909 | 100.00 | 100.00 | 100.00 |
| MG491654 | 1679.72 | 0 | *Serratia sp.* | 909 | 100.00 | 100.00 | 100.00 |
| MG491653 | 1679.72 | 0 | *Serratia sp.* | 909 | 100.00 | 100.00 | 100.00 |
| MG491652 | 1679.72 | 0 | *Serratia sp.* | 909 | 100.00 | 100.00 | 100.00 |
| MG491651 | 1679.72 | 0 | *Serratia sp.* | 909 | 100.00 | 100.00 | 100.00 |
| MG491546 | 1679.72 | 0 | *Serratia sp.* | 909 | 100.00 | 100.00 | 100.00 |
| MG491534 | 1679.72 | 0 | *Serratia sp.* | 909 | 100.00 | 100.00 | 100.00 |
| MG491533 | 1679.72 | 0 | *Serratia sp.* | 909 | 100.00 | 100.00 | 100.00 |
| KY780233 | 1679.72 | 0 | *Serratia sp.* | 909 | 100.00 | 100.00 | 100.00 |
| KY780232 | 1679.72 | 0 | *Pseudomonas sp.* | 909 | 100.00 | 100.00 | 100.00 |
| KY612275 | 1679.72 | 0 | *Serratia marcescens* | 909 | 100.00 | 100.00 | 100.00 |
| KY421549 | 1679.72 | 0 | *Serratia marcescens* | 909 | 100.00 | 100.00 | 100.00 |
| KX373971 | 1679.72 | 0 | *Serratia sp.* | 909 | 100.00 | 100.00 | 100.00 |
| KU145656 | 1679.72 | 0 | *Serratia sp.* | 909 | 100.00 | 100.00 | 100.00 |
| KP340289 | 1679.72 | 0 | *Serratia sp.* | 909 | 100.00 | 100.00 | 100.00 |
| KM021144 | 1679.72 | 0 | *Serratia sp.* | 909 | 100.00 | 100.00 | 100.00 |
| KJ672309 | 1679.72 | 0 | *Serratia marcescens* | 909 | 100.00 | 100.00 | 100.00 |

**Table S4.** The 20 best hits from a BLAST using the *Providencia rettgeri* consensus sequence of clones 60ED55, 60EI20 and 61CD10 (partial 16*S* SSU rRNA gene sequence).

| **Accession** | **Bit-Score** | **E Value** | **Organism** | **Sequence Length** | **% Pairwise Identity** | **% Identical Sites** | **% Query coverage** |
| --- | --- | --- | --- | --- | --- | --- | --- |
| KY744946 | 1749.9 | 0 | *Providencia rettgeri* | 947 | 100.00 | 100.00 | 100.00 |
| KX289656 | 1749.9 | 0 | *Providencia rettgeri* | 947 | 100.00 | 100.00 | 100.00 |
| KF193115 | 1749.9 | 0 | Bacterium endosymbiont | 947 | 100.00 | 100.00 | 100.00 |
| KF193111 | 1749.9 | 0 | Bacterium endosymbiont | 947 | 100.00 | 100.00 | 100.00 |
| GU193984 | 1749.9 | 0 | *Providencia rettgeri* | 947 | 100.00 | 100.00 | 100.00 |
| GU166190 | 1749.9 | 0 | Uncultured bacterium | 947 | 100.00 | 100.00 | 100.00 |
| GQ417423 | 1749.9 | 0 | Uncultured Providencia | 947 | 100.00 | 100.00 | 100.00 |
| GQ417411 | 1749.9 | 0 | Uncultured Providencia | 947 | 100.00 | 100.00 | 100.00 |
| KJ004633 | 1744.36 | 0 | *Providencia rettgeri* | 947 | 99.90 | 99.90 | 100.00 |
| KF712889 | 1744.36 | 0 | *Providencia sp.* | 947 | 99.90 | 99.90 | 100.00 |
| KC456595 | 1744.36 | 0 | *Providencia rettgeri* | 947 | 99.90 | 99.90 | 100.00 |
| KC456569 | 1744.36 | 0 | *Providencia rettgeri* | 947 | 99.90 | 99.90 | 100.00 |
| KC456540 | 1744.36 | 0 | *Providencia rettgeri* | 947 | 99.90 | 99.90 | 100.00 |
| JX000034 | 1744.36 | 0 | Uncultured bacterium | 947 | 99.90 | 99.90 | 100.00 |
| HQ407257 | 1744.36 | 0 | *Providencia rettgeri* | 947 | 99.90 | 99.90 | 100.00 |
| GU457413 | 1744.36 | 0 | *Providencia rettgeri* | 947 | 99.90 | 99.90 | 100.00 |
| EU660370 | 1744.36 | 0 | *Providencia rettgeri* | 947 | 99.90 | 99.90 | 100.00 |
| EU660367 | 1744.36 | 0 | *Providencia rettgeri* | 947 | 99.90 | 99.90 | 100.00 |
| AY870456 | 1744.36 | 0 | *Providencia sp.* | 947 | 99.90 | 99.90 | 100.00 |
| MF099655 | 1738.82 | 0 | *Providencia sp.* | 947 | 99.80 | 99.80 | 100.00 |

**Table S5.** Summary of the Kaplan-Meier survival analysis, including bacterial concentrations, sexes, treatments, number of individuals (n), number of dead individuals during the 10 dpi (events), and ST_50_-values (median) with 95% lower and upper confidence limits (0.95LCL, 0.95UCL).

| **concentration** | **sex** | **treatment** | **n** | **events** | **median** | **0.95LCL** | **0.95UCL** |
| --- | --- | --- | --- | --- | --- | --- | --- |
| 8 × 10^9^ cells/ml | females | PBS | 15 | 0 | NA | NA | NA |
|  |  | *S. sciuri* | 15 | 8 | 156 | 96 | NA |
|  |  | *P. rettgeri* | 15 | 15 | 36 | 24 | 36 |
|  |  | *S. marcescens* | 15 | 15 | 24 | NA | NA |
|  | males | PBS | 15 | 0 | NA | NA | NA |
|  |  | *S. sciuri* | 15 | 3 | NA | NA | NA |
|  |  | *P. rettgeri* | 15 | 15 | 36 | 24 | 36 |
|  |  | *S. marcescens* | 15 | 15 | 24 | 24 | 24 |
| 8 × 10^7^ cells/ml | females | PBS | 15 | 0 | NA | NA | NA |
|  |  | *S. sciuri* | 15 | 0 | NA | NA | NA |
|  |  | *P. rettgeri* | 15 | 15 | 60 | 60 | 84 |
|  |  | *S. marcescens* | 15 | 14 | 60 | 48 | 96 |
|  | males | PBS | 15 | 0 | NA | NA | NA |
|  |  | *S. sciuri* | 15 | 0 | NA | NA | NA |
|  |  | *P. rettgeri* | 15 | 12 | 60 | 60 | NA |
|  |  | *S. marcescens* | 15 | 12 | 60 | 48 | NA |

**Table S6.** Results of the Kaplan-Meier survival analysis, using log-rank test with Holm-corrected p-values to investigate differences between treatments (comparison) for both bacterial concentrations (8 × 10^9^ cells/ml and 8 × 10^7^ cells/ml) and sexes (females and males). Significant p-values are depicted in bold.

| **comparison** | **8 × 10^9^ cells/ml** | | **8 × 10^7^ cells/ml** | |
| --- | --- | --- | --- | --- |
|  | **females** | **males** | **females** | **males** |
| *S. sciuri* vs PBS | **2.13 × 10^-03^** | 7.26 × 10^-02^ | 1.00 | 1.00 |
| *P. rettgeri* vs PBS | **6.25 × 10^-08^** | **1.10 × 10^-07^** | **8.55 × 10^-08^** | **3.40 × 10^-05^** |
| *S. marcescens* vs PBS | **2.90 × 10^-07^** | **1.10 × 10^-07^** | **4.64 × 10^-07^** | **3.40 × 10^-05^** |
| *P. rettgeri* vs *S. sciuri* | **6.25 × 10^-08^** | **1.21 × 10^-07^** | **8.55 × 10^-08^** | **3.40 × 10^-05^** |
| *S. marcenscens* vs *S. sciuri* | **2.90 × 10^-07^** | **1.10 × 10^-07^** | **4.64 × 10^-07^** | **3.40 × 10^-05^** |
| *S. marcenscens* vs *P. rettgeri* | **2.13 × 10^-03^** | **1.20 × 10^-02^** | 1.00 | 1.00 |

**Table S7.** Results of the Kaplan-Meier survival analysis, using log-rank test with Holm-corrected p-values to investigate pairwise differences between females and males within the different treatments for both bacterial concentrations tested in the survival experiments. Significant p-values are depicted in bold.

| **comparison** | **concentration** | **treatment** | **p-values** |
| --- | --- | --- | --- |
| female vs male beetles | 8 × 10^9^ cells/ml | PBS | 1.0000 |
|  |  | *S. sciuri* | 0.0539 |
|  |  | *P. rettgeri* | 0.9207 |
|  |  | *S. marcescens* | 0.3173 |
|  | 8 × 10^7^ cells/ml | PBS | 1.0000 |
|  |  | *S. sciuri* | 1.0000 |
|  |  | *P. rettgeri* | 0.1713 |
|  |  | *S. marcescens* | 0.4155 |

**Table S8.** List of 23 gene-specific primer pairs used for AMP gene expression analysis (22 AMPs and RPS3 as housekeeping gene for normalization of qPCR data) adopted from our previous study (Gegner *et al.*, 2008; https://doi.org/10.1038/s41598-018-21781-4).

| **Primer** | **Target gene** | **Sequence forward (5’-3’)** | **Sequence reward (5’-3’)** |
| --- | --- | --- | --- |
| Att4 | Attacin4 | CAAGAATCCAGGAGGTACTCAAG | ACCGCCTCCTAATGTTGTTG |
| Att6 | Attacin6 | AAGAATCCAGGAGGTACACAGG | ACCGCCTCCTAATGTTGTTG |
| Att10 | Attacin10 | CGCTTTAGCCTCTTCAGAATCA | TCCTTTATGCCCCAAAGTCA |
| Att18 | Attacin18 | ACCCACAAGGTACGCAGGT | CCTCCTCCTAATGCTGTTGG |
| Col1 | Coleoptericin1 | CTGCATCTCCTTCCAATATGC | GGTCCTTCGGGAACAACATA |
| Col5 | Coleoptericin5 | CATTTGCCTGCATTTACTTCC | TTGGGGCTCCAGGTAGAAG |
| Col8 | Coleoptericin8 | GGAAGAGGTTGTTGTAGATGGAG | TCTGGTGAGTGATGGGTCAA |
| ColLA | Coleoptericin-likeA | GAAGGATGGCAAGTCGAACA | ACCACGTGAAGGTTCCTCCT |
| ColLB | Coleoptericin-likeB | AGATGTGCCTTGCTTCGAGA | ATTGTGCGAGAACCACCAAC |
| ColLC | Coleoptericin-likeC | CATGTTCGGGACTCTGAAGG | CTCACAGATCCGGCAGTGTT |
| ColLD | Coleoptericin-likeD | CACGTTCGGGATACTGATGG | CACCTGAAGGTTGCTCCAAG |
| CLys1 | C-type Lysozyme1 | GCGTTTGTGCAGAGAAGGTC | TAACATGGCGGAAGTGGAAG |
| CLys2 | C-type Lysozyme2 | TCAAAGCTTGGAATGGTTGG | TCCATCAACATAGCGGCATT |
| CLys3 | C-type Lysozyme3 | GGGAATTCCAGGCAATCAGT | CATGGCAACCTTTACCAGGA |
| CLys4 | C-type Lysozyme4 | GGGTCAGAAGCCCGAATTTA | CAGCGGTGTCAAAGTTGGAT |
| Def1 | Defensin1 | CATTCTCCTCTCCTACCGAACC | GGCGACACAATGTAATGCAC |
| Def3 | Defensin3 | ACCGAAAGGGGAAATTCTTC | GGCGACACAATGTAATGCAC |
| Def7 | Defensin7 | TGAGTATCGAAGCAGGAACCA | TCCTCCCTCGTAGTTGAAGG |
| SapL7 | Sapecin-like Defensin7 | ACCGAACCGAAAGAGGAAAT | GCAGCTTAAAGCACATGCAG |
| Thau1 | Thaumatin1 | TTCTGGGGAAGAAGTTGGTG | CTCGACAAGGGTAGCTGGAG |
| Thau2 | Thaumatin2 | TGAAGGTGGACAAGTGTGGA | CAGGGGCATAAACAGAGACC |
| Thau4 | Thaumatin4 | AGGGCTGGACTTCTACGACA | TCCTGGACAATGGTTGTTGA |
| RPS3 | Ribosomal proteine RPS3 | GGCTACCAGAACCGACAGAG | GTGCTATGGCGCATAATCCT |

**Table S9.** Results of the parametric multiple comparison analysis on qPCR control data. A one-way ANOVA model was used for each of the 22 measured AMP genes with ΔCT as response variable and replicate as factor to test for pairwise differences between the three control replicates (n = 6 each) within time points (24h and 48h replicates) and between time points (24 vs. 48h replicates).

| model factor | Att4 | Att6 | Att10 | Att18 | CLys1 | CLys2 | CLys3 | CLys4 | Col1 | Col5 | Col8 |
| --- | --- | --- | --- | --- | --- | --- | --- | --- | --- | --- | --- |
| 24h replicates | 0.2580 | 0.9420 | 0.0504 | **0.0324** | 0.0663 | 0.3310 | **0.0451** | 0.6750 | **0.0348** | 0.3280 | **0.0267** |
| 48h replicates | 0.2580 | 0.9420 | 0.5230 | 0.2740 | 0.0663 | 0.3310 | **0.0451** | 0.6750 | **0.0206** | 0.0568 | **0.0498** |
| 24 vs. 48h replicates | 0.4530 | 0.1510 | 0.0594 | **0.0155** | 0.1220 | **0.0190** | 0.0757 | 0.6780 | **0.0117** | **0.0442** | **0.0110** |
|  |  |  |  |  |  |  |  |  |  |  |  |
| model factor | ColLA | ColLB | ColLC | ColLD | Def1 | Def3 | Def7 | SapL7 | Thau1 | Thau2 | Thau4 |
| 24h replicates | **0.0028** | 0.8500 | **0.0300** | 0.1900 | 0.6250 | 0.8090 | 0.1270 | 0.9900 | 0.2210 | 0.9170 | 0.2490 |
| 48h replicates | **0.0028** | 0.8500 | **0.0300** | 0.1900 | 0.6250 | 0.8090 | 0.1270 | 0.9900 | 0.2210 | 0.9170 | 0.2490 |
| 24 vs. 48h replicates | 0.1570 | **0.0040** | **0.0178** | 0.0590 | 0.1180 | 0.4190 | 0.7600 | 0.9430 | 0.2130 | 0.5750 | 0.3490 |

Significance level after p-value adjustment for multiple testing using the heteroscedasticity robust sandwich estimator for the covariance matrix is p < 0.05. Significant values are depicted in bold.

**Figure S1.** *Parasitylenchus bifurcatus* consensus sequence from the *de novo* assembly of PCR products representing the partial 18*S* SSU rRNA gene sequence.

CTCAAAGATTAAGCCATGCATGTCTAAGTATAAACGAATCAATCGTGAAACCGCGAACGGCTCATTAAATCAGCTATGATCTACTTGATCTTGAGACTACTCCTACTTGGATAACTGTGGTAATTCTRGAGCTAATACATGCACAAAAGCCCTGACCTCGCAGGAGGGGCGCATTTATTAGAGCAAAACCAGGCGACCTTGGTCGTTGTTGCTGACTCAGAATAACTCAGCTGATCGCACGGTCTCGTACCGGCGACGTGTCTTTTGAGTATCTGCCTTATCAATTTTCGATGGTAGTGTATTTGACTACCATGGTTTTGACGGGTAACGGAGAATCAGGGTTCGACTCCGGAGAAGGGGCCCGAGAAATGGCCACTACGTCTAAGGATGGCAGCAGGCGCGCAAATTACCCACTCTCGGAATGAGGAGGTAGTGACGAGAAATAACGAGACCGTTCTCTATGAGGCCGGTCATCGGAATGGGTACAATCTAAACCCTTTAACGAGTATCTATGAGAGGGCAAGTCTGGTGCCAGCAGCCGCGGTAATTCCAGCTCTCAAAGTGCATAGAACCATTGTTGCGGTTAAAAAGCTCGTAGTTGAATCTAGGTCTGAGGCCTGGTCCACACACTGTGTGCGCACTAGGTTCTTTGGCTTTGACGTCGGTTTGCTTGTCGTCGCCTTTACCGGCGGCGGCCGGTGGCTGGCAATTTTACTTTGAACAAATCAAAGTGCTCAAAACAGGCGTTTCGCTTGTATGCTTTTGCATGGAATAATAGAATATGACTTCGGTCTGGTTTTATTGGTTTTATAGATCGTTGTAATGATTAACAGGGTCAAGCGGGGGCATTCGTATTGTTGCGTGAGAGGTGAAATTCTTGGACCGCAGCAAGACGACCAACAGCGAAAGCATTTGCCAAGCTTGTCTTCATTAATCAAGAACGAAAGTCAGAGGATCGAAGGTGATCAGATACCGCCCTAGTTCTGACCGTAAACGATGCCAACTAGCGATTCGCCGGCGGATTTTTTGCCCTGGCGAGGAGCTTCCCGGAAACGAAAGTCTTCCGGTTCCGGGGGAAGTATGGTTGCAAAGCTGAAACTTAAAGGAATTGACGGAAGGGCACCACCAGGAGTGGAGCCTGCGGCTTAATTTGACTCAACACGGGAAAACTCACCCGGCCCGGACACTAGGAGGATTGACCGATTGATAGCTCTTTCATGATTTGGTGGATGGTGGTGCATGGCCGTTCTTAGTTCGTGGAGTGATTTGTCTGGTTTATTCCGATAACGAGCGAGACTTTAGCCTACTAAATAGTCCGCACATTATACTCTATTGTGTAGCGGTACTTCCTAGAGGAATTGTCGGTGTTTAACCGCAAGAAAGTGAGCAATAACAGGTCTGTGATGCCCTTAGATGTCCGGGGCTGCACGCGCGCTACACTGGTAAAGTCAGCGTGCATGTCCTTCCTCGAAAGAGGTTGGTAAACCGATGAAAGTTTGTCGTGATTGGGATCGGAGATTGCAATTATTTTCTGTGAACGAGGAATTCCAAGTAAATGTGAGTCATCAACTTGCGTTGATTACGTCCCTGCCCTTTGTACACACCGCCCGTCGCTGCCCGGAACTGAGCCATTTCGAGAAAGCTGGGGATTGCTGATCGACGGTTCTACGGGAATCGTCTTTTGGTGAAAACCAGTTTAATCGTGGTGGCTTGAACCGGGCAAAAGTCGTAACAAGGTAGC

**Figure S2.** *Staphylococcus sciuri* consensus sequence of clones 60ED58, 60ED89 and 60EI10 (partial 16*S* SSU rRNA gene sequence).

AGCTTGCTTCTCTGATGTTAGCGGCGGACGGGTGAGTAACACGTGGGTAACCTACCTATAAGACTGGGATAACTCCGGGAAACCGGGGCTAATACCGGATAATATTTTGAACCGCATGGTTCAATAGTGAAAGACGGTTTCGGCTGTCACTTATAGATGGACCCGCGCCGTATTAGCTAGTTGGTAAGGTAATGGCTTACCAAGGCGACGATACGTAGCCGACCTGAGAGGGTGATCGGCCACACTGGAACTGAGACACGGTCCAGACTCCTACGGGAGGCAGCAGTAGGGAATCTTCCGCAATGGGCGAAAGCCTGACGGAGCAACGCCGCGTGAGTGATGAAGGTCTTCGGATCGTAAAACTCTGTTGTTAGGGAAGAACAAATTTGTTAGTAACTGAACAAGTCTTGACGGTACCTAACCAGAAAGCCACGGCTAACTACGTGCCAGCAGCCGCGGTAATACGTAGGTGGCAAGCGTTATCCGGAATTATTGGGCGTAAAGCGCGCGTAGGCGGTTTCTTAAGTCTGATGTGAAAGCCCACGGCTCAACCGTGGAGGGTCATTGGAAACTGGGAAACTTGAGTGCAGAAGAGGAGAGTGGAATTCCATGTGTAGCGGTGAAATGCGCAGAGATATGGAGGAACACCAGTGGCGAAGGCGGCTCTCTGGTCTGTAACTGACGCTGATGTGCGAAAGCGTGGGGATCAAACAGGATTAGATACCCTGGTAGTCCACGCCGTAAACGATGAGTGCTAAGTGTTAGGGGGTTTCCGCCCCTTAGTGCTGCAGCTAACGCATTAAGCACTCCGCCTGGGGAGTACGACCGCAAGGTTGAAACTCAAAGGAATTGACGGGGACCCGCACAAGCGGTGGAGCATGTGGTTTAATTCGAAGCAACGCGAAGAACCTTACCAAATCTTGACATCCTTTGACCGCT

**Figure S3.** *Serratia marcescens* consensus sequence of clones 60ED57, 60EI15 and 61CD07 (partial 16*S* SSU rRNA gene sequence).

CGGCGGACGGGTGAGTAATGTCTGGGAAACTGCCTGATGGAGGGGGATAACTACTGGAAACGGTAGCTAATACCGCATAACGTCGCAAGACCAAAGAGGGGGACCTTCGGGCCTCTTGCCATCAGATGTGCCCAGATGGGATTAGCTAGTAGGTGGGGTAATGGCTCACCTAGGCGACGATCCCTAGCTGGTCTGAGAGGATGACCAGCCACACTGGAACTGAGACACGGTCCAGACTCCTACGGGAGGCAGCAGTGGGGAATATTGCACAATGGGCGCAAGCCTGATGCAGCCATGCCGCGTGTGTGAAGAAGGCCTTCGGGTTGTAAAGCACTTTCAGCGAGGAGGAAGGTGGTGAACTTAATACGCTCATCAATTGACGTTACTCGCAGAAGAAGCACCGGCTAACTCCGTGCCAGCAGCCGCGGTAATACGGAGGGTGCAAGCGTTAATCGGAATTACTGGGCGTAAAGCGCACGCAGGCGGTTTGTTAAGTCAGATGTGAAATCCCCGGGCTCAACCTGGGAACTGCATTTGAAACTGGCAAGCTAGAGTCTCGTAGAGGGGGGTAGAATTCCAGGTGTAGCGGTGAAATGCGTAGAGATCTGGAGGAATACCGGTGGCGAAGGCGGCCCCCTGGACGAAGACTGACGCTCAGGTGCGAAAGCGTGGGGAGCAAACAGGATTAGATACCCTGGTAGTCCACGCTGTAAACGATGTCGATTTGGAGGTTGTGCCCTTGAGGCGTGGCTTCCGGAGCTAACGCGTTAAATCGACCGCCTGGGGAGTACGGCCGCAAGGTTAAAACTCAAATGAATTGACGGGGGCCCGCACAAGCGGTGGAGCATGTGGTTTAATTCGATGCAACGCGAAGAACCTTACCTACTCTTGACATCCAGAGAACTTTCC

**Figure S4.** *Providencia rettgeri* consensus sequence of clones 60ED55, 60EI20 and 61CD10 (partial 16*S* SSU rRNA gene sequence).

AGTAATGTATGGGGATCTGCCCGATAGAGGGGGATAACTACTGGAAACGGTAGCTAATACCGCATAATCTCTCAGGAGCAAAGCAGGGGAACTTCGGTCCTTGCGCTATCGGATGAACCCATATGGGATTAGCTAGTAGGTGAGGTAATGGCTCACCTAGGCGACGATCCCTAGCTGGTCTGAGAGGATGATCAGCCACACTGGGACTGAGACACGGCCCAGACTCCTACGGGAGGCAGCAGTGGGGAATATTGCACAATGGGCGCAAGCCTGATGCAGCCATGCCGCGTGTATGAAGAAGGCCCTAGGGTTGTAAAGTACTTTCAGTCGGGAGGAAGGCGTTGATGCTAATATCATCAACGATTGACGTTACCGACAGAAGAAGCACCGGCTAACTCCGTGCCAGCAGCCGCGGTAATACGGAGGGTGCAAGCGTTAATCGGAATTACTGGGCGTAAAGCGCACGCAGGCGGTTGATTAAGTTAGATGTGAAATCCCCGGGCTTAACCTGGGAATGGCATCTAAGACTGGTCAGCTAGAGTCTTGTAGAGGGGGGTAGAATTCCATGTGTAGCGGTGAAATGCGTAGAGATGTGGAGGAATACCGGTGGCGAAGGCGGCCCCCTGGACAAAGACTGACGCTCAGGTGCGAAAGCGTGGGGAGCAAACAGGATTAGATACCCTGGTAGTCCACGCTGTAAACGATGTCGATTTGAAGGTTGTTCCCTAGAGGAGTGGCTTTCGGAGCTAACGCGTTAAATCGACCGCCTGGGGAGTACGGCCGCAAGGTTAAAACTCAAATGAATTGACGGGGGCCCGCACAAGCGGTGGAGCATGTGGTTTAATTCGATGCAACGCGAAGAACCTTACCTACTCTTGACATCCAGAGAACTTAGCAGAGATGCTTTGGTGCCTTCGGGAACTCTGAGACAGGTGCTGCATGGCTGT

**Figure S5**. *Providencia sp.* 16*S* SSU rRNA gene sequence dendrogram. Position of the newly isolated *P. rettgeri* (consensus) is shown among other described *Providencia* species. *Moellerella wisconsensis* served as outgroup. Branch labels show units of substitutions per site of the sequence alignment.

**Figure S6**. Quantification of nematode numbers in the suspensions used for the infection of *H. axyridis* beetles in our AMP gene expression analysis. We analysed a still image from a quick time movie we made during the dissection process. In the frame analysed below, we used the multi-point tool in ImageJ 1.51 j8 and counted 296 infective juveniles (IJ). The depicted binocular frame covered 25% of the whole sample (200µl), thus 1.184 IJ/200µl (4 x 296=1.184). Finally, as the nematode suspensions had about 1 ml volume, the concentration for infections was approximately 6000 nematodes/ml (5 x 1.184=5920).


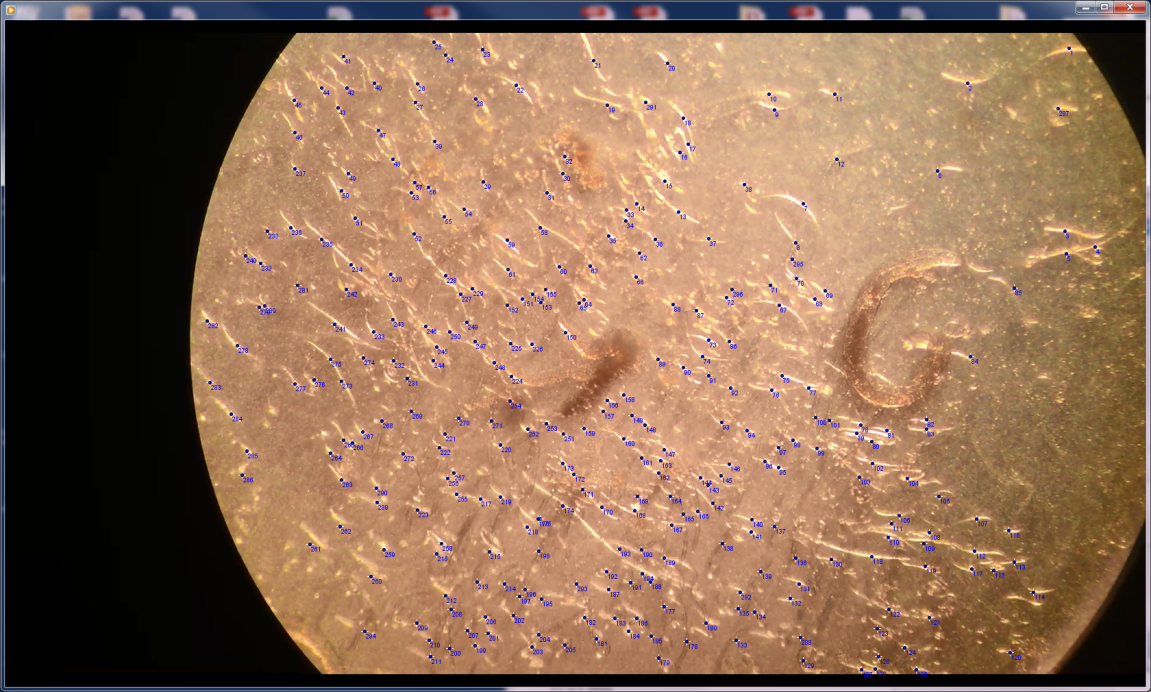

Supplement: Supplementary file 1 — Supplementary Information [file 41598_2018_34278_MOESM1_ESM.docx]
